# “We Don't Feel so Alone”: A Qualitative Study of Virtual Memory Cafés to Support Social Connectedness Among Individuals Living With Dementia and Care Partners During COVID-19

**DOI:** 10.3389/fpubh.2021.660144

**Published:** 2021-05-13

**Authors:** Sara S. Masoud, Kylie N. Meyer, Lauryn Martin Sweet, Patricia J. Prado, Carole L. White

**Affiliations:** ^1^Caring for the Caregiver, School of Nursing, University of Texas Health Science Center at San Antonio, San Antonio, TX, United States; ^2^School of Health Professions, University of Texas Health Science Center at San Antonio, San Antonio, TX, United States

**Keywords:** Memory Cafés, dementia, caregiving, social connectedness, isolation, COVID-19

## Abstract

**Introduction:** Loneliness and low social support can be detrimental to the health of individuals living with Alzheimer's and related dementias (ADRD) and family care partners. Restrictions on gatherings to prevent the spread of COVID-19 create an even greater risk for social isolation. Memory Cafés are a highly replicated program that provide individuals living with ADRD and care partners an opportunity to socialize in an inclusive and supportive environment without fear of judgment, pressure, or stigma. Following restrictions on in-person gatherings, virtual Memory Cafés offer regular social engagement opportunities in an online format. While the Memory Café model has been replicated globally, their effects on loneliness and perceived social support are generally unknown. Even less is known about their impact when operating in a virtual environment.

**Methods:** Semi-structured interviews in Spanish and English were conducted with individuals living with dementia and family care partners who regularly attend Memory Cafés hosted by partners in a Texas Memory Café Network. Interviews took place online using video conferencing software, were transcribed, then analyzed for common themes using a combined inductive and deductive approach.

**Results:** A total of 17 interviews were conducted with persons living with dementia (*n* = 5) and family care partners (*n* = 12) who attend Memory Cafés to learn about their perceived experiences of social connectedness since COVID-19. Care partners included spouses (*n* = 8) and adult children (*n* = 4). Interviews included attendees of different Memory Café models, including in-person only (*n* = 2), virtual only (*n* = 9), and those who attend both models (*n* = 6). Five key themes were identified: (1) Reprieve; (2) What is still possible; (3) Connectedness; (4) Inclusivity; and (5) Value added, with ten sub-themes supporting these main themes.

**Discussion:** Findings substantiate evidence that Memory Cafés offer important benefits for families living with dementia, providing vital new insight into the potential for virtual Memory Cafés to offer similar benefits. Findings have implications beyond the context of COVID-19, where virtual models may support the social connectedness of those living in geographically marginalized and underserved areas. Virtual models may not address the needs of all families experiencing dementia due to lack of access to technology and limitations for virtual engagement with those experiencing later stage dementia.

## Introduction

In the U.S., there are ~5.8 million people 65 years and older living with Alzheimer's dementia (AD), with numbers expected to reach 7.1 million by 2025 and 13.8 million by 2050 (1). Persons living with dementia and their family care partners often experience detrimental social consequences, including higher risk for loneliness and diminished social support (2, 3). Stigma around dementia contributes to isolation and poor social connectedness for both the individual and their care partners, such as when care partners avoid telling others about the condition (4). Loneliness and low social support can have negative consequences for the health of persons living with AD and their care partners. Low social support and loneliness are associated with poorer cardiovascular, immune, and mental health, and has been shown to increase the risk of dementia (5–8). Furthermore, research has shown that individuals with low social support are at an increased risk of mortality (9). Persons living with dementia and their care partners often experience a reduction in size of their social networks and loss of connection with others as the disease progresses (10).

COVID-19 restrictions on gatherings intended to mitigate viral spread put older adults living with dementia and family care partners at even greater risk for social isolation (11–13). To date, the consequences of the pandemic on the progression of dementia remain unknown, although there is evidence linking social isolation and the exacerbation of dementia symptoms (14). As families living with dementia are further disconnected due to these safety restrictions, they are also navigating fears and anxieties around infection for themselves and their family member living with dementia (15). COVID-19 has resulted in mass closures of community programs for older adults including day programs and senior centers. These closures have decreased opportunities for older adults to connect socially and have left care partners responsible for the social and cognitive engagement of persons living with dementia (16, 17). There is currently no cure or effective pharmacological treatment to prevent or reverse AD. As such, there is a need for non-pharmacological approaches to address the social impacts of the condition, particularly following COVID-19 social distancing measures.

Memory Cafés, sometimes referred to as Alzheimer's or Dementia Cafés, are a widely implemented program that provide individuals living with dementia and their care partners an opportunity to socialize with others. These spaces, whether virtual or in-person, provide individuals living with dementia a place to socialize without fear of stigma or judgment due to behavioral symptoms of their diagnosis (18–20). The experiences of persons living with dementia at all stages are often focused on their cognitive impairment, rather than on the strengths and abilities that still remain (4). Memory Cafés focus on the capacities that individuals living with dementia maintain, including the ability to connect with others, express themselves creatively, and participate in group activities. For care partners, these programs allow them to socialize with their family member, rather than just focusing on care-related responsibilities, and to connect with other caregivers (18, 19).

While the Memory Café model has been highly replicated across the globe—making them potentially more accessible than many existing service interventions—little is known about how they may affect the social connectedness of attendees and even less is understood of their impact in a virtual context. There is evidence that group-based interventions lower loneliness and increase social support among older adults, although this predominantly describes effects of complex, multi-component programs rather than community-based programs like the Memory Café model (3, 21). Further, studies to describe the impact of Memory Café on participants have predominantly focused on the experiences and perspectives of family care partners and coordinators (18, 22). This exploratory qualitative study provides valuable insight into the experiences of individuals living with dementia and family care partners who regularly attend Memory Cafés, and explores how these gatherings affect perceptions of social connectedness.

## Methods

Using a qualitative descriptive design, we interviewed persons living with dementia and family care partners who regularly participate in Memory Cafés to better understand their experiences related to perceived social connectedness. Participants were eligible for interviews if they (1) self-identified as being a person living with dementia or a care partner to a person living with dementia; (2) exhibited capacity to consent to the study and discuss their experiences in interviews; (3) had attended at least two Memory Café events; (4) could participate over the telephone or video conferencing; and (5) spoke either English or Spanish. Participants were recruited using attendance sheets collected from three Memory Cafés in the Texas Memory Café Network (TMCN). An invitation with study details was emailed to attendees of the three Memory Café sites. Eligibility was verified for those who responded with interest to participate in interviews.

The three Memory Cafés from which participants were invited for this study all host monthly gatherings online using video conferencing software (two use Zoom and one uses Microsoft Teams). As Memory Cafés should adapt to the needs and interests of their attendees, each site has a unique approach to the logistics and planning of their gatherings. However, all three sites follow some core principles encouraged by the TMCN such as hosting Memory Café gatherings at least once a month, training all staff and volunteers to be dementia inclusive, taking pre-registration for each gathering, planning activities that are accessible and respectful of individuals living with dementia. One Memory Café is facilitated by a memory care center, enabling residents to connect with their family members who were unable to visit them during COVID-19 restrictions. This Memory Café is also open to non-resident community members. Two of the participating Memory Café sites were in-person and transitioned to virtual and the other site established their virtual Memory Café shortly after the pandemic was declared. One Memory Café hosts their gatherings in a bilingual English and Spanish format and is open for registration without geographic restrictions, often promoting their gatherings via local and national networks. The other two Memory Cafés target their promotion to encourage engagement at a local level though they still welcome attendees from other cities if there is interest. As members of the TMCN, these sites share access to resources to support planning inclusive and engaging activities for families living with dementia. Some of the activities hosted at these Memory Café gatherings include “creative conversations,” music and movie trivia, making strawberry jam, Lotería (Mexican bingo), and guest facilitators including medical clowns, a music therapist, and non-profit art programs.

The study protocol was submitted to the Institutional Review Board (IRB) of UT Health San Antonio and provided with approval prior to study initiation. The requirement for written consent was waived by the IRB. All participants were provided with verbal and written information sheets about the project (available in English and Spanish) and verbal consent was obtained upon recruitment and again at the start of each interview. Interviews were conducted by three individuals who were experienced engaging with individuals living with dementia and family care partners for research and events. One interviewer facilitates a Memory Café and as such only interviewed attendees of other Memory Cafés to limit response bias. Two interviewers were graduate research assistants, one of them bilingual in English and Spanish. Interviewers were knowledgeable about dementia and, although no formal tool was used to assess capacity to assent, were prepared to discontinue interviews if responses from persons living with dementia indicated poor understanding of the interview purpose or unwillingness to participate at any time during the interview.

A thorough review of existing literature about Memory Cafés (in-person and virtual) was conducted to inform the development of the field note and interview guides. A list of keywords was developed and periodically refined to assist two graduate research assistant in identifying literature about Memory Cafés. Citations were compiled and reviewed by the project Co-PIs and research assistants over several meetings, the keywords were updated as new literature was collected. From reviewing the literature, a criteria checklist was generated to list key characteristics of Memory Cafés, distinguished by the required criteria to fit the Memory Café description and some common, but not necessary criteria. These criteria were used to generate a field note guide for observation of the virtual models offered by the three participating Memory Café sites in the TMCN.

Field note observations were taken by the project Co-PI and another member of the research team at a total of five virtual Memory Café events hosted by the three sites. One of the Co-PIs is the host of a participating Memory Café site and did not take observations at their own site. Field notes were assessed to determine which of the criteria were observed at each site and observe what approaches were used to engage participants and how they seemed to respond to the activities. The research team reviewed the notes to identify engagement strategies used in the virtual Memory Cafés, potential challenges to engagement, and how engaged participants were at each Memory Café event. These preliminary field note observations, the literature review findings, and the criteria checklist were used to develop an interview guide.

Interview guides were designed to understand the social connectedness experiences of individuals who attend in-person and/or virtual Memory Cafés. The initial interview guide was modified slightly after the first two interviews were completed to improve clarity and flow. The interview guide can be accessed as a Supplementary Material. Interviews were conducted online via Zoom or over the telephone, depending on participant preference. Persons living with dementia were asked if they would prefer to participate in interviews with or without their care partners in the same room. If care partners were present for interviews with persons living with dementia, they were instructed to refrain from answering on behalf of the person living with dementia. The length of interviews ranged from ~30 min to an hour and a half. All interviews were recorded and transcribed verbatim. As one of the Memory Cafés hosts events in a bilingual format, monolingual Spanish speakers were also invited to participate in the interviews. Spanish transcriptions were translated to English and the interviewer was consulted to verify for cultural translation in meaning.

### Data Analysis

Data were analyzed using a combined inductive and deductive approach. Initial coding was informed by broader themes derived from the observational field notes and literature review. The research team took efforts to enhance the quality of coding analysis through a process of researcher triangulation to define codes and make decisions in building the codebook. After the first interview was completed, two researchers independently read the transcription and assigned codes, using the initial themes as a guiding framework and then compared codes to refine a final thematic framework for analyzing the remaining transcripts. To support the credibility and dependability of the study, the research team met regularly at all phases of the project and coders met weekly to review codes during the analysis phase. All transcripts were analyzed by two independent coders. Two additional researchers were regularly consulted to review emerging themes and for resolution of any coding discrepancies between the two coders. The full team reviewed final themes and definitions along with corresponding data to ensure the perspectives and experiences of participants were captured. Finally, themes were presented to the Texas Memory Café Network for their insight as to whether they felt the findings resonated with their experiences coordinating Memory Cafés.

## Results

A total of 17 interviews were conducted for this exploratory study and as data saturation was reached, no further participants were interviewed. All except one of the participants completed interviews via Zoom, the other completed their interview by telephone with no apparent difference in their data. Care partners (*n* = 12) and individuals living with dementia (*n* = 5) who had attended virtual Memory Cafés in the TMCN were interviewed to evaluate their experiences. Two interviews were conducted in Spanish. Care partners included spouses (*n* = 8) and adult children (*n* = 4) of persons living with dementia. Two participants had attended only in-person Memory Cafés and nine had only attended virtual while the remaining six had attended both formats. Although interviews began after the COVID-19 pandemic had begun, funding for this research was awarded prior to the official declaration of the pandemic in March 2020. As such, participants who had only attended in-person Memory Cafés were included in interviews and their responses were included in analysis as their insight was important in comparing the experiences and perspectives of in-person and virtual attendees. While in-person Memory Cafés could often accommodate a wider range of participants at different stages of disease progression, virtual Memory Café activities are primarily focused on those living with early- to mid-stage dementia. Further, consenting individuals living with more advanced stages of dementia to participate in a study poses added challenges. As such, those living with dementia who participated in this study were all experiencing early- to mid-stage dementia. All individuals living with dementia who were interviewed for this study were living with their care partners. Demographics are described in Table 1.

**Table 1:** Participant demographic details.

***N* = 17**	***n* (%)**
Persons living with dementia	5 (29.4)
Care partners	12 (70.6)
Spouse	8 (66.7)
Adult children	4 (33.3)
**Gender**	
Female	11 (64.7)
Male	6 (35.3)
**Language**	
English	15 (88.2)
Spanish	2 (11.8)
**Format**	
In-person only	2 (11.8)
Virtual only	9 (52.9)
Both	6 (35.3)

Five overarching themes were identified from the interviews: (1) Reprieve; (2) What is still possible; (3) Connectedness; (4) Inclusivity; (5) Value added, with ten sub-themes supporting these main themes (Table 2). Selected quotes are used to describe each theme below. Participants are identified by their status (e.g., caregiver), their relationship to the care recipient (e.g., spousal), and what model of Memory Café they attended (e.g., virtual only).

**Table 2:** Themes, subthemes, and key findings.

Reprieve	**Something to look forward to** • Gives something to plan for/anticipate; the opportunity to do something enjoyable each month
	**Break from daily life** • A reprieve from stressors of caregiving or living with dementia, and also a break from COVID-19
What is still possible	• Realizing what is still possible when living with dementia or caregiving for someone who is living with dementia • Growing together as a family after the diagnosis (the experience of living with dementia gives them the opportunity to continue connecting and learning about each other in novel ways)
Connectedness	**Feeling of Belonging** • Fitting in, feeling like a “part of it” (i.e. the Memory Café community)
	**Sense of community** • Communicating with people from shared experiences outside their households • Supporting relationships beyond the context of the Memory Café
Inclusivity	**Accessibility** • Structuring programs to support user-friendliness for participants of different abilities • Planning around attendee suggestions, needs, & interests • Using technology facilitates inclusion for some and excludes others from participating • Dementia-aware staff are important for attendees to feel relaxed, secure, and comfortable
	**Diversity** • Create the opportunity for Spanish-speaking families to attend • Sharing cultural traditions and heritage with others
Value Added	**Cognitive stimulation** • Engaging in activities to support brain health
	**Education** • Receiving education from activity facilitators and peers
	**Resources** • Learning about available community resources and services
	**Helping others** • Suggesting activities for the events to engage others in their families and communities • Seeing the benefits of Memory Cafés on beyond their own personal experiences

### Theme (I) Reprieve

The majority of interviewees expressed that Memory Cafés offer some sense of reprieve from their typical day-to-day experiences as individuals living with dementia or care partners, and also from the unique stressors following COVID-19 safety precautions. This theme was particularly strong for participants who have attended in-person Memory Cafés, although it was a consistent theme for virtual attendees as well.

#### Something to Look Forward to

Memory Café attendees feel anticipation for upcoming events. Family care partners in particular shared that they appreciate the opportunity to plan to do something enjoyable each month.

“*It gives us a better—**something to look forward to*** *than the ordinary, daily things that you have to do on a daily basis.”* – Spousal care partner, virtual only

“*Memory Café is once a month. It's not like it's a commitment every week or two times a week or five hours when it is done. It's like, no. It's something to look forward to.”* – Adult child care partner, both models

“*It puts* ***something to do for today**. Something to look forward to.”* – Person living with dementia, both models

#### Break From Daily Life

Attendees welcome the opportunity to do something different from their usual routines and day-to-day lives. Care partners shared that Memory Cafés permit them to “let go a little bit” and enjoy the chance to participate in activities alongside their family member living with dementia.

“*I think it's most especially with this pandemic that we're having and we're having to stay home*, ***this is a little outlet**. Although we don't leave the house, it's something different. It's something that you're able to just—I don't wanna call it a release, but it gives a little bit of* ***something different to do**.”* – Spousal care partner living with dementia, virtual only

“*Being together, but also for me personally, it gives me okay*, ***I can let go a little bit***…* It gives me a break for the 30 minutes or for the hour. It's important that we, as a caregiver, have. Yeah without leaving him at a daycare or having to take him to some place”* – Spousal care partner, both models

“***We've been locked up because of the quarantine**. Goin' out and goin' to the memory café gives you that opportunity [to get a break from quarantine].”* – Person living with dementia, virtual only

### Theme (II) What Is Still Possible

Some participants expressed that the activities remind them of what they continue to be capable of despite living with dementia. Several participants reflected that Memory Cafés facilitate meaningful moments and the opportunity to connect in new ways with their family members such as the chance to learn about each other.

“*My experience with the Memory Café has given [me] a short outlet to get away from the fact that I have a disability and to be able to play loter*í*a [bingo] like a—not like a professional, but you know what I'm sayin' like a normal person and be able to do these activities*. ***It gives me a source of accomplishment that wow. I could do that. I did it***.” – Person living with dementia, virtual only

“*You get to know people and recognize*, ***even if I can't remember the name, I recognize*** ***them and say ‘hi' and know where they're at***. *It's odd. I can't remember the name, but I remember—I know what they're gonna say or whatever 'cause we've met ‘em before*.” – Person living with dementia, both models

“*The one that I attended with my mom showed me that she can still interact, and she wants to. That was a benefit, versus some of the stuff you read*, ***it's almost like once a person*** ***gets diagnosed, it's almost like you're supposed to put them in the background*** ***and not take them out anymore. It's like, no, she can still interact***. *We just have to find the right venue for her and for me*.” – Adult child care partner, both models

### Theme (III) Connectedness

A sense of connectedness was reflected by attendees of both models who shared that they felt, or desired to feel, a sense of belonging and to build a community among the group. Some participants shared challenges and identified barriers to connectedness in the virtual environment. Several participants expressed that they felt it would take more time to develop their connectedness than it would in-person yet they were interested in continuing despite these challenges.

#### Feeling of Belonging

A goal of Memory Cafés is to facilitate a sense of belonging amongst attendees and most interviewees reported feeling they are an included and welcomed member of the group. Some participants, however, expressed that they did not feel as though they were part of a group of peers at the Memory Cafés. Barriers were attributed to the virtual context and needing more time to get to know others through the Memory Café.

“*I get to talk to other caregivers. He gets to talk to people in his situation, too, and so the two of us—**it helps because we don't feel so alone, so isolated I guess***, ***and the understanding of what we're going through each one of us when we*** ***go into the groups, we just—okay, we're accepted**.”* – Spousal care partner, both models

“***It makes me feel connected**. It makes me feel a part and of whatever it is we're doin'…”* – Person living with dementia, virtual only

“*I don't feel like I do about the support group, which I've been to for much longer and, also, in person and done some socializing with some of them*. ***It'll take a*** ***while, I think, to feel that we're part of a group**.”* – Spousal care partner, virtual only

#### Sense of Community

Participants shared sentiments that indicated a sense of community between attendees can be facilitated by the Memory Cafés. This is stimulated by the chance to communicate with others outside their households and the opportunity to cultivate their relationships with others outside of the Memory Cafés.

“*I think* ***it's the social part that it's helped with**. I would have to say that's the biggie. You're not so isolated.”* – Person living with dementia, both models

“*We attend because not only does it help the person living with dementia, it helps the caregiver, and I guess that… it gives [my spouse]* ***the opportunity to interact with others,*** ***and it gives me a time to, yes, interact with others**.”* – Spousal care partner, both models

“*She feels related to or familiarized virtually with the people that she has met in person. For example, she attends her events and she is able to recognize her friends online*. ***This is the first time that we are in a program where the introduction and*** ***the only method of seeing each other is virtually***…* I think we will have a better answer in a year because it is a different experience.”* – Adult child care partner, virtual only (speaking about their parent's connectedness online following COVID-19)

### Theme (IV) Inclusivity

Participants expressed sentiments around accessibility and diversity in relation to having a sense of inclusivity at virtual Memory Cafés. Accessibility has been attributed to the structure and logistics of Memory Cafés, emphasizing planning, coordination, and receptiveness to attendee feedback. Technology is an important factor in determining whether participants felt included in the virtual Memory Café model. In-person attendees shared insight into why they are no longer able or interested in attending in the virtual context. Diversity is another theme that contributed to feeling included, many attendees having attended a bilingual Memory Café shared how that component motivated them to participate.

#### Accessibility

Accessibility is a significant contributing factor to experiences of inclusivity shared by participants. Interviews reflected that attendees feel the Memory Cafés are responsive to their interests and recommendations for planning activities and logistics such as meeting days and times.

“*Of course, it has been a great connection with the coordinators too… and they really do understand the context of each person. I have also given them very strong opinions, but I think it is important to share with honesty because there might be other needs that the program might have*. ***They seem extremely open to opinions from new participants**.”* – Adult child care partner, virtual only

While several participants shared that the use of technology for virtual Memory Cafés is a welcomed and often preferable change, others are excluded from participation. This is attributed by some to an aversion to the virtual mode of engagement, lack of access or unfamiliarity with the technology, or the inability to meaningfully engage with others in a virtual capacity.

“*We've enjoyed the [virtual] Memory Café as is. I do know that mom and dad—it is more likely that they will attend via the Zoom than if it was in person… because to get mom out of the house, it becomes an ordeal*.” – Adult child care partner, virtual only

“*If we were just meeting—if it was meeting in a location and not virtually, I'm sure they would be probably seen a little bit differently and the interaction would be different. Probably that would be more beneficial, but certainly* ***this has served its purpose for what*** ***is needed in this society right now the way it is**.” –* Spousal care partner, virtual only

“*These are good but it's not the same.”* – Spousal care partner (when asked why they chose not to attend virtual Memory Cafés), in-person only

“*For the Memory Cafe, but that's another thing my husband's memory is getting worse. I'm not even sure he'd be able to—and his vision is also getting worse*. ***we're not sure of his capabilities right now**.”* – Spousal care partner, in-person only

Further, accessibility of Memory Cafés can be attributed to staff training. Participants placed importance on the knowledge and training of staff in dementia-friendly behaviors such as planning appropriate events, accommodating the needs of attendees living with dementia, and understanding dementia inclusive strategies for communication.

“*One of the things is that y'all are experts*. ***Y'all don't talk down to us***. *Y'all talk to us and we're able—I feel able to confident to pick up my hand and give an opinion.” –* Person living with dementia, virtual only

#### Diversity

The inclusivity of Memory Cafés in terms of diversity in culture, heritage, and language is important to some participants. They reflected that they enjoyed learning about the cultures of other attendees and sharing their personal customs with others and appreciated when planned activities encouraged this practice. Participants of a bilingual Spanish/English Memory Café shared that they appreciated the unique opportunity to engage in their preferred language. Monolingual attendees did not find the bilingual format disruptive and often appreciated the opportunity to learn new words in a different language.

“*So, we are really glad that we found this Bilingual program and* ***we appreciate that they*** ***will translate for us because she feels more included***…” – Adult child care partner, virtual only

“*I feel right at home with — I think it's just wonderful*. ***Why not be bilingual**?*” – Spousal care partner (in reference to bilingual format), virtual only

### Theme (V) Value Added

While the predominant purpose of Memory Cafés is to socialize with others, attendees shared several other benefits of participation.

#### Cognitive Stimulation

Many sought the cognitive stimulation that comes with socializing with others and engagement in planned activities like bingo or sing-alongs.

“***It's keepin' our brain functioning**, and so, I think yes, definitely, that this should be somethin' that should continue on.”* – Person living with dementia, virtual only

“*We're always looking for ways to keep active and keep social, so in that way, that's one of the rules of being a caregiver is to try and find those experiences and* ***keep the brain working and stimulated**.”* – Spousal care partner, virtual only

#### Education

Several shared the Memory Cafés were a space where they could receive education from facilitators or health professionals in attendance and also from observing and conversing with peers.

“*That has helped because* ***I understand more in seeing other people, how they deal with*** ***their, the person who has it***. *It gives me that experience.”* – Adult child care partner, both models

“*You will be dealing with professionals that work with this every day that can* ***give you*** ***the kind of information that you need***…* Now, my kids and my husband know what there is out there because of our goin' to the Memory Café.”* – Person living with dementia, virtual only

#### Resources

Resource sharing was a significant benefit for several attendees, many expressed a desire to learn about what is available in the community for dementia and caregiving support.

“*There are other caregivers going through the same thing*. ***We're trying to figure out how*** ***do we get groceries**,' cause you get tired of just bein' in the house but is it safe to go out to even get groceries?”* – Adult child care partner, both models

“*I think that even the Memory Café is an important part of the preparation because we have—if we're stuck with somethin'*, ***we know we have someplace that we can*** ***call*** *or to ask a question or whatever.”* – Person living with dementia, virtual only

#### Helping Others

Another added benefit to participation in Memory Cafés is the sense that they can help others by suggesting engaging activities, sharing their knowledge and experiences with others, and encouraging individuals in their own networks to give Memory Cafés a chance so they can experience the benefits directly.

“*To me*, ***the Memory Caf*****é** ***can do a lot for the community*** *as far as supporting the people that are goin' through this trauma and as well as supporting the family members that are havin' to deal with it too.”* – Person living with dementia, virtual only

“*I think that it is helpful definitely for the caregiver and allows the person that you are caring for to see other people in the same situation and to be able to participate in activities”* – Spousal care partner, both

## Discussion

This study explored the impact of virtual Memory Cafés on the social connectedness experiences of persons living with dementia and family care partners in the midst of the COVID-19 pandemic. Findings suggest that virtual Memory Cafés may be effective facilitators of social connectedness for families living with dementia. Key themes from this study are in line with existing evidence that Memory Cafés may be beneficial to participants in supporting their socialization needs as well as providing some additional benefits for attendees (18, 20, 23). Of particular importance following restrictions on social gatherings since COVID-19, this study sheds some light on how virtual models of Memory Cafés might achieve these established benefits of these community programs.

Two individuals interviewed for this study had only attended in-person Memory Cafés, providing insight into the perspectives of those who were unable or uninterested in participating in the virtual model. While the key themes are reflective of participant experiences of both in-person and virtual Memory Cafés, some areas resonated more strongly within certain groups. Two themes, *feeling of belonging* and *accessibility*, reflected notably divergent perspectives among those who attended virtually and those who had attended only in-person Memory Cafés or both formats. Participants who attended in-person events shared stronger sentiments reflecting a sense of belonging, potentially signifying that the virtual approach may not be as conducive to achieving this goal. While the feeling of fitting in was stronger among attendees who had also attended in-person models, virtual Memory Café participants did still reflect that they were able to meaningfully connect with their family member(s) as well as others outside their households, despite barriers to physical contact. This finding substantiates other studies of support programs that have reported that care partners desire connectedness with others who have commonalities in their carer experiences (24, 25). Coordinators of virtual Memory Cafés can aim to plan activities that focus on facilitating interactions between households, not just between the host(s) and guests. Interviews indicate that virtual models may have some limitations in terms of accessibility to facilitate a sense of belonging, although they may still meet an important need for care partners to be connected to others with shared experiences.

The ability to continue to socialize is a priority for participants, many who felt they preferred the virtual option, as they were previously not able to attend in-person events due to caregiving responsibilities, schedule conflicts, or distance. This supports findings from other studies that identified similar barriers to connectedness for family care partners of persons living with dementia outside the context of the COVID-19 pandemic, which has exacerbated these barriers for many (26–28). Participants who had attended both formats were able to give important insight into the benefits of each model, some expressing a preference for in-person connection while acknowledging that the virtual option provides needed support and social connection when in-person is not available. As socialization is the primary goal of Memory Cafés, it is significant that findings suggest meaningful social connection is attainable in a virtual environment among the study population.

Our findings substantiate results from evaluations of in-person models showing that while most Memory Café guests are primarily motivated to attend to connect with others, many guests seek more than social opportunities and also hope to meet a range of other needs (18, 23). In supporting the social well-being of families living with dementia, our findings indicate that virtual Memory Cafés can also address other practical needs, including the opportunity to learn through observing others in attendance, engaging in cognitively stimulating activities, and being connected to community resources. While findings reveal that virtual models can be effective in facilitating these added advantages, there seems to be a need for increased intentionality in the planning and implementation of these online programs in order to facilitate these benefits (e.g., using visual cues for those living with a memory impairment). Table 3 outlines by theme some recommendations for Memory Café coordinators to consider when organizing their events.

**Table 3:** Recommendations for coordinators by theme.

**Theme**	**Subthemes**	**Recommendations for coordinators**
Reprieve	• Something to look forward to • Break from daily life	• Get to know your guests so you can plan activities that can build anticipation and hopefulness between events (example: Lotería). • Implement marketing and promotion strategies that emphasize the opportunity to break routine in a non-disruptive way.
What is still possible	• Capacities and opportunities that remain	• Set guests up for success by planning activities that play to their strengths (e.g., reminiscence).
Connectedness	• Feeling of Belonging • Sense of community	• Aim to facilitate interactions between guests, not just between hosts and guests. • Encourage guests to connect outside of the Memory Café through email, social media, or telephone.
Inclusivity	• Accessibility • Diversity	• Invest in teaching guests the basic functionalities of your hosting platform. Accommodate guests using different technology to access (computer, tablets, smartphones). • Consider those who are not there. Invest in checking in on families without technology to join and identify barriers to access. • Commit to the language needs in your community. Consider inviting bilingual facilitators or hiring translation support. • Create culturally inclusive spaces by modeling sharing, facilitating conversations, and encouraging storytelling.
Value Added	• Cognitive stimulation • Education • Resources • Helping others	• Guests attend for more than just socialization. Plan activities that promote cognitive stimulation, conversation, and resource sharing. • Invite one or two community partners to join as active participants so guests can familiarize and build relationships with local resources. • Guests often have altruistic desires to help their family members and communities. Empower guests by encouraging them to share their ideas and interests for future activities.

There are some limitations to who and how virtual Memory Cafés support the connectedness of attendees. As reliance on technology has increased since COVID-19, the digital exclusion of older adults who are not connected to technology and internet has become an even more pressing concern (29). Our findings reflect elements of digital exclusion that are exacerbated by dementia and the caregiving role. Some family caregivers who only attended Memory Cafés in-person felt they were left out of participating following the transition to virtual platforms. Several participants expressed that the virtual format is not accessible to their family member living with dementia due to behavioral symptoms or that they have a general disinterest in using the technology, and many simply do not have access to reliable internet or computers. Coordinators can take extra steps to support these families by checking in via phone and assisting them in finding potential solutions to barriers or identifying alternative options for social engagement (e.g., telephone-based programs).

Key themes reflect that while virtual Memory Cafés have strengths and weaknesses related to accessibility, they may still meet an important need for social connectedness among persons living with dementia and family care partners. Findings support evidence that Memory Cafés are beneficial for family care partners and persons living with dementia, and the virtual approach may provide an effective alternative to in-person models. These findings have implications beyond the context of COVID-19, where virtual models may support the social connectedness needs of families living with dementia living in geographically marginalized and underserved communities.

### Study Limitations

This study has some limitations. While the sample may seem small given the inclusion of both people living with dementia and family caregivers, however, the richness and depth of the interviews helped to achieve redundancy in thematic analysis. Further, care partners were often able to speak to the experiences of their family member living with dementia who attend Memory Cafés with them, giving added insight into the range of participant experiences (30). It was important to include the perspectives of individuals living with dementia and care partners as the Memory Café model is designed to support both roles. Further, some interviews with persons living with dementia were conducted with the family caregiver present, and thus data from these participants may be subject to response bias. The need to conduct interviews virtually to prevent spread of COVID-19 made it difficult to conduct one-on-one interviews with persons living with dementia, who typically needed support to use videoconferencing technology. In addition, the researchers who conducted this study also hosts a Memory Café which several respondents attended. Here again response bias is a concern given that participants may have shared a more positive experience with Memory Café than they would have otherwise. We partially addressed this concern by having a trained research assistant not involved in conducting the Memory Café conduct interviews. Further, we identified several negative assessments of the virtual Memory Cafés in participants responses, indicating that participants felt comfortable to share negative feedback. Lastly, Memory Cafés do not require guests to disclose personal information like details around their diagnosis or age. This is to support their comfort to attend without fear of stigma or judgment and to provide them the opportunity to share their personal information if and when they choose. With this in mind, we kept our demographic questions for this study to what we felt necessary for our analysis. Memory Cafés are typically planned to support the social connectedness of those living with early- to mid-stage dementia and there are challenges associated with consenting individuals living with more advanced dementia, especially in the virtual environments. As such, we did not ask participants to disclose their time since diagnosis for this study. Despite these limitations, the insights from this exploratory study establish a framework for future evaluation to understand how virtual and in-person models of Memory Café programs can support the social connectedness of persons living with dementia and family care partners. Little is understood about the specific mechanisms that influence the social experiences of Memory Café guests, particularly for persons living with dementia. Although this study represents the perspectives of families living with dementia, there is a need for further investigation into the impact of Memory Cafés centered on the experiences of individuals living with dementia.

### Conclusion

This qualitative study identified virtual Memory Cafés as a potential mechanism to address the social connectedness needs of persons living with dementia and family care partners. Guests attend for social opportunities but anticipate meeting other needs in the process. Coordinators can integrate this into their planning and implementation to provide more impactful programs and activities. Future research should further investigate the experiences of persons living with dementia who attend Memory Cafés, and quantitatively evaluate the extent to which participation improves the health and quality of life of participants.

## Data Availability Statement

The raw data supporting the conclusions of this article will be made available by the authors, without undue reservation.

## Ethics Statement

The studies involving human participants were reviewed and approved by Office of the Institutional Review Board, University of Texas Health Science Center at San Antonio. Written informed consent for participation was not required for this study in accordance with the national legislation and the institutional requirements.

## Author Contributions

KM, SM, and CW contributed to conception and design of the study. SM organized the data base. All authors participated in the data analysis with primary coding conducted by SM and LM. SM wrote the first draft of the manuscript. All authors wrote sections of the manuscript and contributed to manuscript revision, read, and approved the submitted version.

## Conflict of Interest

The authors declare that the research was conducted in the absence of any commercial or financial relationships that could be construed as a potential conflict of interest.
