# Supplementary material for: “We Don't Feel so Alone”: A Qualitative Study of Virtual Memory Cafés to Support Social Connectedness Among Individuals Living With Dementia and Care Partners During COVID-19
Source: Front Public Health. 2021 May 13;9:660144. doi: 10.3389/fpubh.2021.660144 (PMC8155306; doi:10.3389/fpubh.2021.660144)
Supplement: Supplementary file 1 [file Data_Sheet_1.PDF]

Virtual Memory Café interview guide (updated for COVID-19)

1. How long have you been attending Memory Café(s)?

2. How did you first hear about Memory Cafés?

3. Can you tell me why you first decided to attend a Memory Café?

4. What do you enjoy most about attending Memory Cafés?

5. Are there things you don't enjoy or would like to change about the café?

6. Do you usually attend the Memory Café with someone else? If yes, with whom?
   a. Do you think the person you attend with enjoy(s) the Memory Café?

7. Do you feel like you are a part of the Memory Café group? [If need further explanation: Do you feel a sense of *belonging* at the Memory Cafés?]

8. Do you think that attending the Memory Café has had benefits for you? If so, can you describe these benefits.

9. Do you think there are any downsides to attending Memory Cafés? If so, can you describe these benefits.

10. How does the day of the week and the time work for you in attending the memory café?

11. [For people who have been attending virtually] How has the virtual memory café supported you during COVID-19?

12. [For people who have been attending virtually] Has the virtual memory café decreased your sense of social isolation related to Coronavirus restrictions on socialization? Please explain how or how not.

13. [For people who have been attending virtually] Do you feel connected to other people in the online Zoom environment?
    a. If so, what is it that makes you feel connected?
    b. If not, what would you recommend helping you feel more connected over Zoom?

14. Has attending Memory Cafés influenced other parts of your life (whether they were in-person or virtual)?
    [If needed, prompt with examples: their relationships, social connections outside of the Memory Café, social empowerment, stigmatization, etc.]

15. Do you think there should be more opportunities like Memory Cafés in Texas?

16. What advice would you give to someone looking to attend a Memory Café for the first time?

17. Is there anything else you would like to add about your experience with Memory Cafés?
