# Supplementary material for: “We Don't Feel so Alone”: A Qualitative Study of Virtual Memory Cafés to Support Social Connectedness Among Individuals Living With Dementia and Care Partners During COVID-19
Source: Front Public Health. 2021 May 13;9:660144. doi: 10.3389/fpubh.2021.660144 (PMC8155306; doi:10.3389/fpubh.2021.660144)
Supplement: Supplementary file 2 [file Data_Sheet_2.PDF]

# Guía para entrevistas de Memory Café en línea (Revisión COVID -19)

1. Cuanto tiempo han estado participando el Memory Café?

2. Como es que ústed se enteró del Memory Café?

3. Me podría decir porque ústed decidió participar en el Memory Café inicialmente?

4. Qué es lo que más disfruta al participar en el Memory Café?

5. Hay alguna cosa que le gustaría cambiar o que no disfruta de este evento?

6. Ústed participa en este evento con alguien más? Y sí, ¿con quién?

7. Cree que la persona que participa con ústed también lo disfruta?

8. ¿Siente que usted que pertenece al grupo de Memory Café? [Si necesita más explicación; ¿Siente usted que es parte del grupo?]

9. ¿Ústed cree que el participar en el Memory Café le ha traído beneficios? ¿Si es así, podría describir esos beneficios?

10. ¿Ústed considera que existe alguna desventaja de participar en este evento? Si es así, podría describirlos.

11. Ústed cree que la hora y el día está funcionando con su horario?

12. [Para las personas que participan virtualmente] Como considera que el Memory Café ha contribuido en apoyarlo durante esta pandemía COVID -19.

13. [Para las personas que participan virtualmente] ¿Ústed considera que el participar virtualmente ha ocasionado que se sienta menos aislado socialmente debido a las restricciones de socialización en la pandemía?

14. [Para las personas que participan virtualmente] ¿Ústed se siente relacionado con las personas en este sístema virtual?
    a. Si es así, ¿cuál serían las cosas que lo hacen sentir que existe una relación social con el grupo?
    b. Si no es así, ¿cuáles serían sus observaciones o comentarios para mejorar esta situación?

15. ¿El participar en el Memory Café ha influenciado alguna parte de su vida (ya sea que haya participado en persona o virtualmente)? [Si necesita ayuda, exponga ejemplos: las relaciones, conexiones sociales fuera de el Memory

Café, le ha ayudado a sentirse mas seguro en sus relaciones sociales, con la estigmatización, etc.]

16. ¿Usted considera que deberían existir más oportunidades similares al Memory Café en Texas?

17. ¿Qué consejo le daría a alguien que está considerando participar en el Memory Café por primera vez?

18. ¿Hay alguna otra cosa que usted quisiera añadir acerca de su experiencia con el Memory Café?
